# Laboratory Evolution Assays and Whole-Genome Sequencing for the Development and Safety Evaluation of *Lactobacillus plantarum* With Stable Resistance to Gentamicin

**DOI:** 10.3389/fmicb.2019.01235

**Published:** 2019-06-04

**Authors:** Yushan Dong, Fuxin Zhang, Bini Wang, Jiayuan Gao, Jiangtao Zhang, Yuyu Shao

**Affiliations:** College of Food Engineering and Nutritional Science, Shaanxi Normal University, Xi’an, China

**Keywords:** probiotic, antibiotic resistance, laboratory evolution, whole-genome sequencing, resistant gene

## Abstract

The goal of this work was to use laboratory evolution assays and whole-genome sequencing to develop and test the safety of a probiotic, *Lactobacillus plantarum*, with high-level of resistance to gentamicin. The evolution of *L. plantarum* was evaluated under the selective pressure from gentamicin and subsequently when the selective pressure was removed. After 30 days of selective pressure from gentamicin, the minimum inhibitory concentration (MIC) of *L. plantarum* to gentamicin increased from 4 to 512 μg/mL and remained stable at this level. After removing the selective pressure, the resistance of *L. plantarum* to gentamicin decreased to 64 μg/mL after 20 days, and remained stable thereafter. Although the MIC declined it was still higher than the cut-off value recommended by EFSA, indicating that the acquisition of gentamicin-resistance was an irreversible process. Using whole-genome sequencing, gene mutations were identified in the strains that had undergone selection pressure from gentamicin as well as in the strains where the selection pressure was subsequently removed. Specifically, four non-synonymous mutations were detected including one single nucleotide polymorphism (SNP), one insertion, and two structural variants (SVs), of which the mutations in genes encoding the drug resistance MFS transporter and transcriptional regulator of AraC family were only detected in the strains under selective pressure from gentamicin. The results indicate that these mutations play an important role in increasing the resistant levels of *L. plantarum* to gentamicin. The mobility analysis of mutant genes confirmed that they were not located on mobile elements of the genome of highly resistant *L. plantarum*, indicating that horizontal gene transfer was not possible.

## Introduction

Gentamicin is an aminoglycoside antibiotic that is commonly used in clinical practice (Liu et al., 2017); it could be taken orally and plays an important role in the treatment of bacterial infections (Moore, 2010). However, the frequent and inappropriate use of gentamicin has led to the emergence of resistant bacteria that cannot be controlled by the same antibiotic. Besides, the transfer of antibiotic resistant genes between bacterial species is also common and of particular concern (Lerner et al., 2017). Gentamicin is a broad spectrum antibiotic and when applied, it will target not only the pathogenic bacteria, but also the commensal probiotic bacteria such as *Lactobacillus plantarum.* This bacterial species is frequently used in the production of fermented foods and can reside in the human gut where it maintains the balance of gut microbiota, inhibits pathogenic bacteria, and promotes the absorption of nutrients (Hirose et al., 2006; Vries et al., 2006).

Resistance of wild-type bacterial strains (both pathogens and probiotics) can evolve in a long term by natural selection, but resistance in naturally evolved strains is unpredictable (Allen et al., 2010; Schürch and Van, 2017). In contrast, laboratory evolution methods can be used to monitor the evolution of sensitive strains into resistant strains and the process of mutation under controlled antibiotic pressure (Conrad et al., 2009). The possibility of developing antibiotic-resistant probiotics has received increasing research attention, as they could be potentially used in combination with antibiotics to regulate the gut microbiota (Govender et al., 2016; Zhang et al., 2018). However, for a safe use of resistant probiotic in food, it is important to ensure that the resistant genes they carry cannot be transferred to other species, such as pathogens.

Due to the recent advances in sequencing technologies, whole genome sequencing has become an inexpensive tool for studying evolution of bacterial genomes; genomic changes can be detected over time during laboratory experiments to identify mutant genes (Wang et al., 2017; Zhang et al., 2018; Feng et al., 2019). In this work, a highly resistant *L. plantarum* strain was developed, and whole-genome sequencing was performed to identify the resistant genes present in the genome and to evaluate their transferability. This study provides the data necessary to understand whether the gentamicin-resistant *L. plantarum* strain can be safely used in food and in combination with gentamicin in future application.

## Materials and Methods

### Bacterial Strain and Cultivation Conditions

*Lactobacillus plantarum* ATCC14917 was obtained from the American Type Culture Collection and was activated using de Man, Rogosa and Sharpe (MRS) broth (M1175, Oxoid, Basingstoke, United Kingdom) at 28°C for 24 h. The strain was grown in Lactic acid bacteria Susceptibility Medium (LSM) for antimicrobial susceptibility tests and laboratory evolution; LSM was composed of 10% MRS and 90% ISO-Sensitest broth (CM0473, Oxoid) (ISO and IDF, 2010).

### Baseline Susceptibility of *L. plantarum* ATCC14917 to Gentamicin

The susceptibility of *L. plantarum* ATCC14917 to gentamicin (Sigma) was evaluated using the IS010932/IDF223 standard method (ISO and IDF, 2010). A gentamicin stock solution was prepared based on its potency (677 μg/mg), the active compound in gentamicin sulfate salt; the stock solution was then filter-sterilized (0.22 μm) and stored at -40°C. The range of tested gentamicin concentration was from 0.5 to 256 μg/mL and two-fold dilution series of gentamicin were made to evaluate the MIC. Inoculum of *L. plantarum* was prepared according to the ISO10932/IDF223 (ISO and IDF, 2010). Briefly, the individual colonies were picked and suspended in sterile saline till the optical density (625 nm) of solution reached to 0.2. The inoculum was diluted 500-fold using LSM and then added to the serial 2-fold dilution gentamicin-LSM medium at the same volume, bringing a 1000-fold dilution of inoculum and 2-fold more dilution of gentamicin. The strain was grown in LSM medium without gentamicin as the control. After incubation at 28°C for 48 h, the lowest gentamicin concentration inhibiting apparent growth of *L. plantarum* ATCC14917 was recorded as the MIC. The MIC was determined in triplicate.

### Evolution of *L. plantarum* ATCC14917 in the Presence and Subsequent Absence of Selection Pressure From Gentamicin

The IC50 was used as the initial gentamicin concentration to initiate evolution of *L. plantarum* ATCC14917 under selection pressure by gentamicin to develop the high resistance (Zhang et al., 2018; Feng et al., 2019). Two colonies were separately activated in MRS and 40 μL of inoculum were added to 3.96 mL of LSM supplemented with half maximal inhibitory concentration of gentamicin (Wang et al., 2017; Zhang et al., 2018). The *L. plantarum* was then serially subcultured in fresh LSM broth with gentamicin every 24 h at 28°C and the MIC was determined every 5 days. The gentamicin concentration was increased by 1.5-fold every 5 days until the MIC became stable (Zhang et al., 2018; Feng et al., 2019). Two colonies subcultured in gentamicin-free LSM were used as the control.

To understand the evolution of *L. plantarum* when the selection pressure from gentamicin was removed, the highly resistant strains obtained using the procedure described above were inoculated into LSM without gentamicin and continued to subculture as described above until the MIC became stable.

The MIC of evolved strains during passage was determined and the extensive concentration range of the gentamicin was adapted for increase of MIC in different lineages. The evolved strains were stocked in glycerol solution at -40°C.

### Whole-Genome Sequencing and Bioinformatics Analysis

The strains obtained by repeated passage to a stable state under selection pressure from gentamicin (*n* = 2), and then following removal of the selection pressure (*n* = 2), were subjected to whole-genome sequencing analysis together with the control strains (*n* = 4; 2 controls for passage either under selection pressure or removal of selection pressure). Genomic DNA of each strain in the cultivated broth was extracted using a bacterial DNA extraction kit (DNeasy Blood & Tissue Kit, Qiagen, Valencia, CA, United States) following the manufacturer’s instructions. The purity and integrity of the DNA were analyzed by electrophoresis in 1% agarose gels at 100 V for 40 min. Genomic DNA from the strains was sheared into 350-bp fragments for library preparation and sequenced on an Illumina HiSeq 2500 system with pair-end 150-bp sequencing strategies. Raw reads were analyzed using the CLC Genomics Workbench v11.0.1. The quality control of sequencing data, trimming low quality sequences based on quality control (QC) report, mapping reads to reference (ACCESSION: NZ_ACGZ00000000, GenBank), removing duplicate reads, and detecting mutants were made successively to identify the genetic mutations (SNP, InDel, and SV) presented in lineages. The recognition rate and length fraction were set to 80% and 0.5 respectively to map reads to the reference. Sanger sequencing was performed to confirm the reliable mutations.

To determine the mobility of the mutation site, homology search (Lugli et al., 2016) and Colombo software (Waack et al., 2006) were used in order to analyze mutations of potential resistant genes in the 10 Kb range flanking each gene. Then the mobility of the predicted genes was detected according to the method of Duranti et al. (2016) to determine whether the mutated genes were located in mobile genetic elements.

### Statistical Analysis

The MIC of all strains was determined in triplicate only to ensure the reproducibility. Since the MIC of biological replicates were stable, no standard deviation under the MIC mean is presented. The figures were plotted with Origin8.5.

### Accession Number

The raw paired-end reads with FASTQ format were obtained from base calling using Casava (v1.8.2; Illumina Inc.). All the eight samples sequencing data are deposited in the NCBI Sequence Read Archive (SRA) database under the accession number of PRJNA528414.

## Results

### Resistance of the Original *L. plantarum* to Gentamicin

The MIC of *L. plantarum* ATCC14917 to gentamicin is shown in **Table 1**. Using the microbiological cut-off values defined by EFSA (European Food Safety Authority [EFSA], 2012) which distinguish between resistant and susceptible strains, we found that the MIC of the original *L. plantarum* ATCC14917 was 4 μg/mL. The obtained value is below the cut-off value of 16 μg/mL, signifying that *L. plantarum* ATCC14917 was sensitive to gentamicin.

**Table 1:** The MIC (μg/mL) of *L. plantarum* ATCC14917 to gentamicin prior to the evolution experiment.

Antibiotic	Replicate	Gentamicin Concentration (μg/mL)										Cut-off value (μg/mL)	Drug-resistant type
		256	128	64	32	16	8	4	2	1	0.5		
Gentamicin	1	-	-	-	-	-	-	-	+	+	+	16	S
	2	-	-	-	-	-	-	-	+	+	+		S
	3	-	-	-	-	-	-	-	+	+	+		S


*- represents no growth of the bacterium. + represents growth of the bacterium. Cut-off value (European Food Safety Authority [EFSA], 2012). S, Susceptible*.

### Evolution of *L. plantarum* in the Presence and Subsequent Absence of Selection Pressure From Gentamicin

**FIGURE 1:**
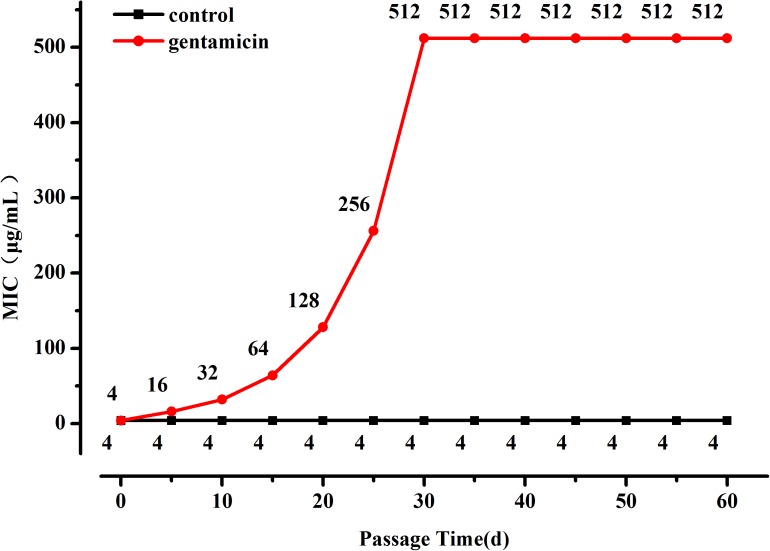
Changes in MIC of *L. plantarum* ATCC14917 to gentamicin over a 60 day period of regular passage in increasing concentrations of gentamicin.

The OD_600_ value of the *L. plantarum* ATCC14917 culture broth with 2 μg/mL of gentamicin was approximately half of the OD_600_ value of culture without gentamicin after cultivating at 28°C for 24 h. Thus evolution of *L. plantarum* ATCC14917 was initiated in the LSM medium containing 2 μg/mL of gentamicin. During subculture the MIC of *L. plantarum* increased progressively but then became stable after 30 days; the MIC of *L. plantarum* under control conditions remained unchanged (**Figure 1**). Over the course of the experiment, the MIC of *L. plantarum* under selection pressure of gentamicin increased from 4 μg/mL at the beginning to 512 μg/mL after 30 days, representing a 128-fold increase in resistance compared with the original strain.

**FIGURE 2:**
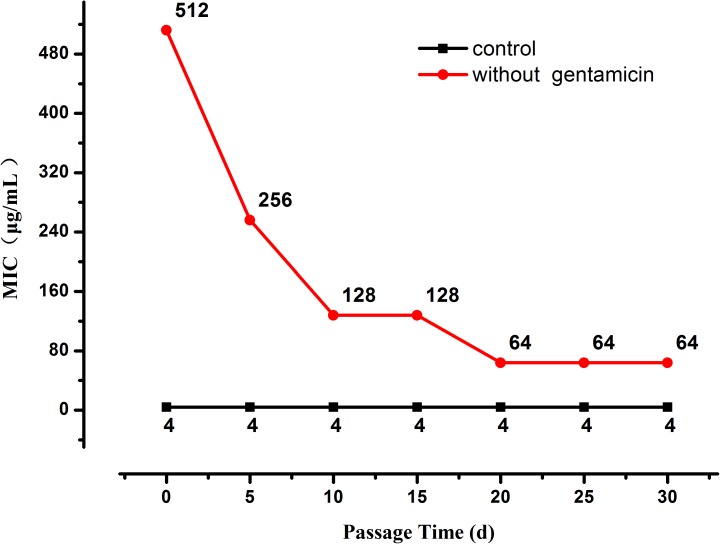
Changes in MIC of resistant *L. plantarum* ATCC14917 in LSM without gentamicin, i.e., when selection pressure from gentamicin is removed.

When the selection pressure from gentamicin was removed the MIC of the highly resistant *L. plantarum* strains decreased progressively until it stabilized at 64 μg/mL after 20 days (**Figure 2**), nevertheless it was still higher than the MIC of the original strain.

### Mutant Genes Analysis

**FIGURE 3:**
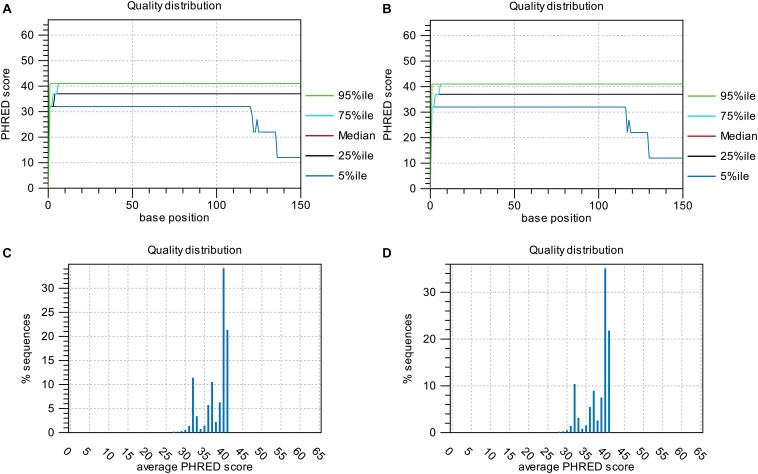
Base-quality distribution along the base positions for sequences of strains following passage under selection pressure from gentamicin **(A)** and following subsequent passage in the absence of gentamicin **(B)**. PHRED scores present the median and percentiles of quality scores observed at different base positions. Distribution of average sequence quality scores for sequences of strains following passage under selection pressure from gentamicin and following subsequent passage in the absence of gentamicin is presented in panels **(C,D)**, respectively. The quality of a sequence is calculated as the arithmetic mean of its base qualities. The sequences observed at different quality scores are normalized to the total number of sequences. The figures were generated using CLC Genomics Workbench 11.0.1.

Base-quality distribution along the base positions and distribution of average sequence quality scores of sequences are presented in **Figure 3**. Bioinformatics analysis showed that four non-synonymous mutations were present in the strains obtained under the selective pressure of gentamicin. These mutations included one SNP and one insertion in two genes regulating hypothetical protein, and two SVs in genes encoding the drug transporter MFS and transcriptional regulator of the AraC family (**Table 2**).

**Table 2:** Mutations detected in *L. plantarum* ATCC14917 following passage under selection pressure from gentamicin and following subsequent passage in the absence of gentamicin, i.e., when selection pressure was removed.

Subculture medium	Chromosome	Region/Site	Type		Variant Length(bp)	Variant ratio	Gene or Locus-tag	Product	Amino acid change	KEGG Orthology
LSM with gentamicin (under selection pressure)	GL379762	355182-355204	SV	Deletion	23	1	*lmrB*	Drug resistance MFS transporter	TGIIIPLT	K18926
		355240-355249			10				DVVM	
	GL379763	280882-280909	SV	Deletion	28	0.68	HMPREF0531_10299	Transcriptional regulator, AraC family	LSSSPNCSSE	K18325
		281102-281123			22				VVVGDNPM	
		281269-281302			34				IDYQREMTVGLLMTEL	
		281402-281406			5				FTY	
		281522-281530			9				LFNK	
	GL379765	297964-297981	Insertion		1	0.57	HMPREF0531_11866	Hypothetical protein	multiple changes	NA
	GL379766	260916	SNP		1	NA	HMPREF0531_12271	Hypothetical protein	p.Ile38Leu	NA
LSM without gentamicin (selection pressure removed)	GL379765	297964-297981	Insertion		1	0.57	HMPREF0531_11866	Hypothetical protein	multiple changes	NA
	GL379766	260916	SNP		1	NA	HMPREF0531_12271	Hypothetical protein	p.Ile38Leu	NA


*SNP: single nucleotide polymorphism. SV: structural variant. Variant length: the number of nucleotide replacement, deletion, or insertion. Variant ratio: This fraction is intended to give a hint toward the zygosity of the InDel. The closer the value to 1, the higher the likelihood that the variant is homozygous. Amino acid change: amino acid(s) replacement or deletion; one insertion introduced in the gene results in multiple changes of amino acids thereafter. NA: not applicable*.

The two mutant genes encoding hypothetical proteins were found in both strains that were subjected to selection pressure from gentamicin and the subsequent strains developing following removal of gentamicin selection pressure. However, the mutations in genes encoding drug resistance, the MFS transporter and the transcriptional regulator of the AraC family, were only detected in the strains obtained under selection pressure from gentamicin and not in the strains subsequently developed when selection pressure from gentamicin was removed. This result suggests that the mutations in the genes encoding MFS transporter and the transcriptional regulator of AraC family played an important role in increasing resistance of *L. plantarum* to gentamicin.

### Potential Mobility of Gentamicin Resistance Associated Mutant Genes

Using homologous analysis we found that no mutant genes were located in mobile elements of the genome of the highly resistant *L. plantarum* strains, indicating that the mutant genes in the highly resistant *L. plantarum* strains cannot be transferred horizontally.

## Discussion

Probiotics are usually sensitive or have low resistance to antibiotics (Połka et al., 2016). The MIC of lactic acid bacteria to gentamicin is generally low because it can readily cross the cell membranes (Danielsen et al., 2004; Elkins and Mullis, 2004). This was confirmed in present study which showed *L. plantarum* ATCC14917 was sensitive to gentamicin. Flórez et al. (2006) found that most of the *L. plantarum* strains tested were susceptible to gentamicin. The emergence of antibiotic resistance is mainly due to the long-term adaptation to the pressure of antibiotics (Davies and Davies, 1996).

Laboratory evolution is an experimental method for exploring evolutionary dynamics and understanding adaptive mechanisms for drug resistance under continuous antibiotic selection pressure (Barrick et al., 2009). However, most of the previous studies have been performed on pathogens (Jahn et al., 2017). *L. plantarum* could gain high level resistance after constant exposure to aminoglycosides (George and Halami, 2017), particularly *L. plantarum* had good adaptability to gentamicin and kanamycin with an increasing of antibiotic concentration during the passage. In this work, a probiotic *L. plantarum* strain was selected to perform a laboratory evolution study to better understand the impact of a gentamicin selective pressure on this bacterial species at whole-genome level. During the evolutionary study, it was found that bacterial resistance to gentamicin increased rapidly in the first 30 days of passage reaching a MIC that was higher than the cut-off value defined by EFSA. This indicates that the strain evolved from being gentamicin-sensitive to gentamicin-resistant within a short period of time. With regard to the strain under subculture in gentamicin-free LSM, constant MIC was maintained throughout. Similarly, during the 20 days of evolution in antibiotic free medium, the resistance of *E. coli* to iodothiocyanate complex and levofloxacin was not developed (Tonoyan et al., 2019). Previous research on changes in Gram-negative bacterial resistance when removing the gentamicin selective pressure showed bacterial resistance decreased (Gerding et al., 1991). Although in the present study when gentamicin challenge was removed the MIC decreased, it stabilized in a value still higher than the gentamicin breakpoint in EFSA guidance, indicating that acquisition of resistance in *L. plantarum* was an irreversible process. This suggests that once resistance has been developed in this strain, it would not revert to its original level.

Whole-genome sequencing could be performed on both strains obtained under selective pressure of gentamicin as well as the strains obtained after removing the selective pressure, to understand and compare the genomic profiles of the strains (Köser et al., 2014). With this tool, one SV in the gene encoding the drug resistance MFS transporter was detected in the strain that was under selective pressure from gentamicin but not in the strain from which selection pressure was subsequently removed. The MFS (major facilitator superfamily) transporter is a superfamily membrane transporter that promotes the movement of small solutes on the cell membrane (Pao et al., 1998). MFS transporters can expel intracellular antibiotics into the environment (Langton et al., 2005). In the genome of *Saccharomyces cerevisiae*, there are more than twenty MFS drug transporters that have positive regulatory effects on commonly used antifungal drugs such as itraconazole, ketoconazole and caspofungin (Costa et al., 2014). It could be inferred that, under selection pressure from gentamicin, *L. plantarum* develops a mutation in the gene encoding MFS transporter which prevents gentamicin from crossing the bacterial cell wall, thereby making the strain resistant. However, after removing the selection pressure, resistance of *L. plantarum* to gentamicin decreased and no mutant gene regulating MFS transporter could be detected. Moreover, we found mutations in genes encoding the transcriptional regulator of the AraC family in the strain developed under selective pressure from gentamicin. Transcriptional regulators of the AraC family are widespread amongst bacteria; they regulate genes with multiple functions, including carbon metabolism and stress responses to virulence (Gallegos et al., 1997). Studies have shown that the AraC family regulator GadX increased multi-drug resistance in *E. coli* by activating expression of multidrug efflux genes (Nishino et al., 2008). Drug efflux pump is a major mechanism of bacterial resistance that protects cells from various exogenous toxic compounds such as antibiotics (Riley et al., 2006). Under selection pressure from gentamicin, transcriptional regulators of the AraC family of *L. plantarum* were activated, which could prevent the entry of gentamicin, and consequently increase the levels of resistance to gentamicin. In addition, there were two hypothetical protein-related gene mutations detected in both strains following selection pressure from gentamicin and the strains from which the selection pressure was removed. Although there is no evidence for the function of these hypothetical proteins, it can still be inferred that hypothetical protein might be related to gentamicin resistance. Further studies are required to confirm this hypothesis by characterization of their function (Nan et al., 2009).

Horizontal gene transfer is accomplished by mobile elements that induce various drug resistance genes, and can be transmitted between bacterium of the same species or even between different species (Brown-Jaque et al., 2015). Transfer of antibiotic resistant genes between species or strains is an important concern regarding the safe use of resistant probiotics combined with antibiotics (Gyles and Boerlin, 2014). This combined therapy could only be applied if the resistance genes carried by probiotics would not be transferred to pathogenic bacteria. In the present study, no mutant genes were located in the mobile elements of the genome, indicating that the resistant mutant genes would not be involved in horizontal transfer and that the highly resistant *L. plantarum* could be safely used for therapeutic purposes in combination with gentamicin.

Resistance of *Lactobacillus* to 14 antibiotics should be detected before their application in food additives according to ISO10932/IDF223 (ISO and IDF, 2010) and European Food Safety Authority [EFSA] (2012); the MIC of these antibiotics against the *Lactobacillus* must be lower than the breakpoint value defined. When *Lactobacillus* is classified as resistance to certain antibiotics, the genetic basis conferring to the antibiotics resistance should be determined to ensure the resistant genes presented could not transfer. Due to the large number of resistant genes and unavailable gene sequences, none of standard methods upon identification of the resistant genes and their transferability have been issued. For this reason, we made current experimental protocol, trying to identify resistant genes and their transferability presented in all of the antibiotics set in the standard documents through whole-genome sequencing. Resistance to each of the antibiotic in *Lactobacillus* must be studied to completely resolve safe concern regarding to their antibiotic resistance, including our previous susceptibility test of streptomycin and present work on gentamicin.

Although both streptomycin and gentamicin belong to aminoglycoside, the changes in resistance of *L. plantarum* to the two antibiotics during the subculture and the gene mutations were different (Zhang et al., 2018). A highly streptomycin-resistant *L. plantarum* (131,072 μg/mL) was obtained and the MIC had reached its upper solubility through laboratory evolution in previous study, while MIC of *L. plantarum* to gentamicin was 512 μg/mL which is less than its solubility limit after subjecting to selective pressure in current work. In addition, resistance of *L. plantarum* to the two antibiotics decreased at different level when removing the selective pressure. Previous research showed mutation in the gene encoding ribosomal protein S12 was detected in the *L. plantarum* strain undergone selective pressure from streptomycin. However, the mutant genes regulating the drug resistance MFS transporter and transcriptional regulator of AraC family were detected when the *L. plantarum* strain was under selective pressure from gentamicin. In fact, gentamicin and streptomycin are 2-deoxystreptamine and non-2-deoxystreptamine aminoglycoside antibiotic respectively (Sylvie and Kristin, 2016). The non-2-deoxystreptamine streptomycin could readily interact with ribosomal protein S12 (Springer et al., 2001) comparing with 2-deoxystreptamine gentamicin, indicating the differences in chemical structure may lead to various mutations in the same strain.

Zhao et al. (2013) found that gentamicin-treated mice showed a significant reduction in bacterial diversity of gut microbiota comparing with the control mice. It was proved that combined use of ampicillin and gentamicin induced a significant change in the evolution of the infant gut microbiota (Fouhy et al., 2012). Imbalance of the gut microbiota could lead to various diseases in human. Thus, the possibility of developing antibiotic-resistant probiotics has received increasing research attention, as they could be potentially used in combination with antibiotics to regulate the gut microbiota. In comparison with streptomycin, gentamicin could be taken orally and plays an important role in the treatment of bacterial infections in the gut which is a good candidate for research on effects of gentamicin and *L. plantarum* combination on gut microbiota. The present work on selection and safety evaluation of a highly gentamicin-resistant *L. plantarum* will be the basis for further study.

Overall, in this study the highly resistant *L. plantarum* strains were obtained through laboratory evolution. Under selection pressure from gentamicin, *L. plantarum* developed high levels of resistance and an eight fold decrease in gentamicin resistance was recorded when selection pressure was removed. Although after removing the selective pressure the MIC declined, it was still higher than that of the original strain, meaning that the acquisition of resistance was an irreversible process. Using whole-genome sequencing, mutations related to the acquisition of *L. plantarum* resistance to gentamicin were found in the genome of the highly resistant strain. Nevertheless, no horizontal transfer of the mutant genes was identified. This research provides a basis for the combined use of a highly resistant *L. plantarum* strain and gentamicin in the treatment of pathogen-induced gut disease and for regulating the balance of the gut microbiota.

## Author Contributions

FZ, YS, and BW contributed to the experimental design. YD, JG, and JZ performed the experiments. YS and YD contributed to the data analysis. YS and YD wrote the manuscript.

## Conflict of Interest Statement

The authors declare that the research was conducted in the absence of any commercial or financial relationships that could be construed as a potential conflict of interest.
